# Optimization of Ultrasonic-Assisted Extraction of Phenolic Compounds and Antioxidant Activity from Araticum Peel Using Response Surface Methodology

**DOI:** 10.3390/plants13182560

**Published:** 2024-09-12

**Authors:** Amanda Cristina Andrade, Felipe Tecchio Borsoi, Ana Sofia Martelli Chaib Saliba, Severino Matias de Alencar, Glaucia Maria Pastore, Henrique Silvano Arruda

**Affiliations:** 1 Department of Food Science and Nutrition (DECAN), School of Food Engineering (FEA), University of Campinas (UNICAMP), Campinas 13083-862, São Paulo, Brazil; andradenut@gmail.com (A.C.A.); felipe.tecchio@gmail.com (F.T.B.); glaupast@unicamp.br (G.M.P.); 2 Department of Agri-Food Industry, Food and Nutrition, Luiz de Queiroz College of Agriculture, University of São Paulo, Piracicaba 13418-900, São Paulo, Brazil; sofiasaliba@usp.br (A.S.M.C.S.); smalencar@usp.br (S.M.d.A.)

**Keywords:** *Annona crassiflora* Mart., Cerrado fruit, bioactive compounds, green extraction, byproducts, flavonoids, procyanidins, reactive oxygen species, central composite rotatable design, Brazilian biodiversity

## Abstract

The peel represents a significant portion of the araticum fruit (about 40%), which becomes waste after its consumption or processing. Previous studies have shown that the araticum peel is rich in phenolic compounds; however, little is known about the ideal conditions for recovering these compounds. Therefore, response surface methodology, using a central composite rotatable design, was employed to optimize the extraction process to maximize the total phenolic compounds (TPCs) and enhance the Trolox equivalent antioxidant capacity (TEAC) from araticum peel. The variables optimized were ethanol concentration (EC; 20–80%, *v/v*), extraction time (ET; 5–45 min), and solid–solvent ratio (SSR; 10–100 mg/mL). Additionally, condensed tannins, antioxidant capacity against synthetic free radicals (TEAC and FRAP) and reactive oxygen species (ROS), and the phenolic compounds profile, were evaluated. Optimum extraction conditions were 50% (*v/v*) ethanol concentration, 5 min of extraction time, and 10 mg/mL solid–solvent ratio. Under these conditions, experimental TPCs and TEAC values were 70.16 mg GAE/g dw and 667.22 µmol TE/g dw, respectively, comparable with predicted models (68.47 mg GAE/g dw for TPCs and 677.04 µmol TE/g dw for TEAC). A high condensed tannins content (76.49 mg CE/g dw) was also observed and 12 phenolic compounds were identified, predominantly flavonoids (97.77%), including procyanidin B2, epicatechin, and catechin as the major compounds. Moreover, a potent antioxidant activity was observed against synthetic free radicals and ROS, especially in scavenging peroxyl and hydroxyl radicals. From this study, we obtained the ideal conditions for recovering phenolic compounds from araticum peel using a simple, fast, sustainable, and effective method, offering a promising opportunity for the management of this plant byproduct.

## 1. Introduction

The Brazilian Cerrado is the country’s second-largest biome, covering about 25% of the national territory [1]. This biome hosts an extensive diversity of vegetation, representing 30% of Brazilian biodiversity, with 11.627 native species and around 44% endemic species [2]. The plants of the Cerrado are naturally adapted to survive under extreme environmental conditions, including high temperatures, low water availability, nutrient-poor soil, high incidence of UV radiation, and insect attacks [2,3]. These adaptations lead to a high synthesis of bioactive phytochemicals to allow plants to resist oxidative stress caused by these conditions, resulting in the development of an interesting bioactive profile. Furthermore, the exotic and unconventional fruit species native to the Cerrado, such as araticum (*Annona crassiflora* Mart.), stand out for their unique sensory characteristics, making it one of the 20 most used species in regional cuisine [2,4].

Araticum, commonly known as marolo, belongs to the Annonaceae family. The fruit can weigh from 0.5 to 4.5 kg, with an oval or rounded shape and a green peel that turns greenish-brown when ripe. Its pulp ranges from white to yellowish with a strong, slightly sweet flavor and a pleasant aroma [5]. Araticum pulp is widely consumed by the local population, both in its natural state and as processed like in ice creams, popsicles, jellies, jams, and juices [6]. After consumption or processing, large amounts of byproducts, particularly the peel, are generated, which accounts for 30–40% of the whole fruit weight [7]. Although often treated as waste and discarded, araticum peel exhibits various biological properties such as antioxidant [5,8,9,10], anti-Alzheimer’s [11], anticancer [1,12], antidiabetic [8], anti-obesity [13], antidyslipidemic [14], hepatoprotective [14,15], anti-inflammatory [16], and antibacterial effects [17]. These properties generally are related to its high content of antioxidant compounds, especially phenolic compounds (particularly epicatechin and catechin and their oligomeric forms (procyanidins), quercetin and kaempferol glycosides, and to a lesser extent, some phenolic acid derivatives) [4,5,10,18].

In this context, araticum peel can be used as a material for obtaining phenolic compounds with potential applications in the food, cosmetic, and pharmaceutical industries, using ecological and safe techniques, such as ultrasound-assisted extraction [10,19]. Ultrasound-assisted extraction is an emerging and cost-effective green technology that accelerates the extraction of plant bioactive compounds through the cavitation effect. This process can destroy the tissues or cells, facilitating the release of phenolic compounds without altering their structure or function, improving extraction yields, and potentially reducing solvent consumption, as well as the temperature and time of the process [20,21]. Although ultrasound-assisted extraction has been used in previous studies to recover phenolic compounds from araticum peel [1,5,10], the extraction parameters remain underexplored, and their individual and synergistic effects on phenolic compounds and their bioactivities are still unclear. As a result, existing studies have not focused on optimizing the extraction variables for phenolic compounds and antioxidants from araticum peel, leaving this area of research as a gap that still needs to be filled.

In addition to the extraction technique, the choice of solvent is crucial, as it can significantly influence the type and quantity of bioactive compound extracted. Factors such as polarity, selectivity, toxicity, environmental considerations, and solubility of the target compounds in the solvent should be considered. Ethanol is a green solvent, low-cost, non-toxic, and safe for human consumption, so it can be recommended for the manufacture of food, cosmetic, and pharmaceutical products [22,23]. Other parameters, including solvent concentration, the solid–solvent ratio, temperature, time, and matrix structure, can also affect the efficiency of phenolic compounds extraction [23]. Therefore, determining these variables is essential for the effective recovery of phenolic compounds and should be evaluated for each type of plant matrix.

Based on the above, optimizing ultrasound-assisted extraction parameters allows for maximizing the recovery of phenolic compounds from araticum peel, promoting the reuse of this byproduct with high bioactive value, reducing organic waste discarded into the environment, and offering new industrial applications with potential health benefits for the consumer. Thus, this study aimed to optimize ethanol concentration, the solid–solvent ratio, and extraction time to maximize the recovery of phenolic compounds and antioxidant activity from araticum peel and evaluate the phenolic profile and antioxidant potential against reactive oxygen species. The study of ultrasound-assisted extraction variables for the recovery of phenolic compounds and antioxidants from araticum peel, as proposed here, fills an existing research gap, as no previous study has optimized these parameters to obtain phenolic-rich extracts with high added value.

## 2. Results and Discussion

### 2.1. Optimization of Extraction Based on Total Phenolic Compounds and Antioxidant Activity

#### 2.1.1. Influence of the Extraction Parameters on TPCs and TEAC

Phenolic compounds are the main secondary metabolites produced by plants in response to environmental stress [24]. These compounds are widely recognized for exhibiting antioxidant [25], antibacterial [26], and anti-inflammatory [27] potential. Thus, the extraction method is a crucial step in recovering and purifying phenolic compounds from vegetable matrices [10,19]. In this context, response surface methodology was employed to optimize ultrasound-assisted extraction conditions to maximize the recovery of phenolic compounds from the araticum peel and their antioxidant activity. Table 1 presents the experimental design, which includes three factors based on the central composite rotatable design (CCRD) model, along with the response variables values for total phenolic compounds (TPCs) and Trolox equivalent antioxidant capacity (TEAC).

The TPCs ranged from 50.34 to 74.48 mg GAE/g dw and TEAC values ranged from 270.04 to 832.43 µmol TE/g dw. The maximum value obtained for both responses was in experiment 3 (32.14% ethanol, 36.9 min, and 28.21 mg/mL). Conversely, the lowest value obtained for TPCs was in experiment 10, when the ethanol concentration was increased to 80.03%, the solid–solvent ratio to 55 mg/mL, and extraction time was decreased to 25 min; the lowest value for TEAC was in experiment 6 (67.86% ethanol, 13.10 min, and 81.79 mg/mL). The central points for both responses presented low variation (<15%), indicating good reproducibility of the process.

The experimental outcomes of TPCs and TEAC analyses constituted the basis of fitting the coded versions of the second-order polynomial regression model are described by Equations (1) and (2), respectively:

TPC (mg GAE/g dw) = 68.58 − 0.87 x_1_ − 4.75 x_1_^2^ + 2.32 x_2_ − 0.55 x_2_^2^ − 2.02 x_3_ − 2.07 x_3_^2^ − 1.32 x_1_ x_2_ + 1.16 x_1_ x_3_ + 2.76 x_2_ x_3_ (1)

TEAC (µmol TE/g dw) = 558.72 − 23.74 x_1_ − 31.19 x_1_^2^ + 12.41 x_2_ + 11.34 x_2_^2^ − 151.28 x_3_ − 35.63 x_3_^2^ − 24.27 x_1_ x_2_− 2.74 x_1_ x_3_ − 16.45 x_2_ x_3_ (2)

where x_1_, x_2_, and x_3_ are the coded values of the independent variables.

The regression coefficients were determined and *p*-value results were used to evaluate the significance of the coefficients in each model (Table 2). The models were remarkably significant for both response variables (*p* < 0.0001). The first-order linear effect was significant (*p* ≤ 0.05) for the solid–solvent ratio (x_3_) for TEAC only, while the second-order quadratic effect was significant (*p* ≤ 0.05) for ethanol concentration (x_1_^2^) for TPCs. In both cases, the model components had a negative effect, demonstrating that an increase in the solid-to-solvent ratio progressively reduces TEAC values and that there is an optimal ethanol concentration, beyond which any increase or decrease results in a reduction in TPCs yields. On the other hand, the interaction effect was not significant (*p* > 0.05) for TPCs or TEAC for any of the independent variables.

According to Hefied et al. [21], the use of analysis of variance (ANOVA) is essential for identifying the effects of various factors on the response and determining whether these effects should be considered. The analysis of variance is a reliable method for evaluating the quality of the adjusted model. The ANOVA of the second-order polynomial models for the TPCs and TEAC (Table 3) shows that the model was significant for TEAC (*p* ≤ 0.05), confirming the good fit of the model for this response variable.

The coefficient of determination (R^2^) measures how well the multiple regression model fits the observed experimental data [28]. The closer the R^2^ value is to 1, the better the experimental model corresponds to the actual data [21]. Bae et al. [29] suggest that the R^2^ of the model should be at least 0.80 to ensure a good fit. The R^2^ values obtained for TPCs and TEAC models were 0.6397 and 0.8352, respectively, indicating the adequacy of the model for TEAC.

**Figure 1:**
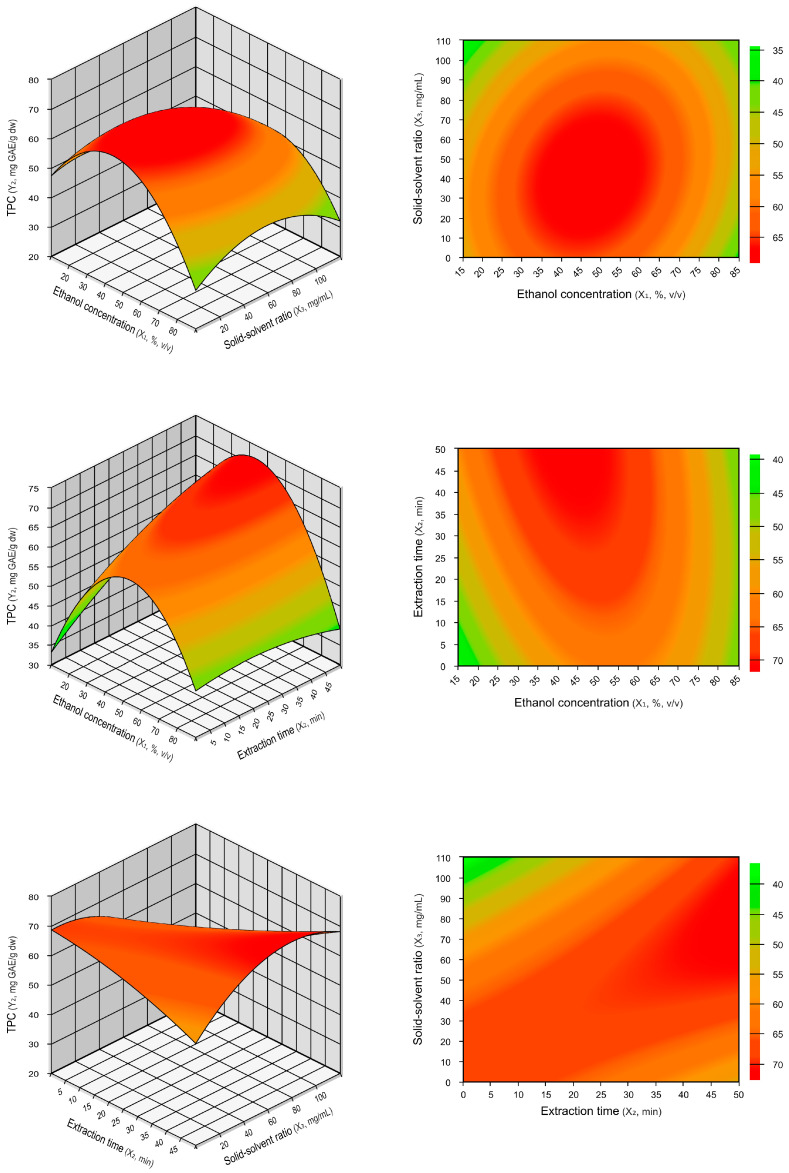
Three-dimensional surface and contour plots illustrating the interaction effects on TPCs extraction based on ethanol concentration (%, *v/v*), extraction time (min), and solid–solvent ratio (mg/mL).

In the surface plot, the steeper the surface, the more pronounced the interaction between the variables. The contour plot is the projection of the response surface onto the bottom plane and represents the interaction between two factors [20]. Figure 1 shows the three-dimensional surface and contour plots of TPCs from araticum peel extract. The results indicate a maximum TPCs with around 50% ethanol concentration. Initially, TPCs recovery increased with increasing ethanol concentration, reaching a maximum level. After this point, when the ethanol concentration exceeded approximately 60%, there was a reduction in TPCs. This result is consistent with Table 2, which shows a significant negative quadratic effect for ethanol concentration.

Özbek et al. [30] demonstrated that an ethanol concentration between 40–50% is usually more effective for extracting phenolic compounds when compared to pure ethanol. Other studies have also reported higher yields of phenolic compound extraction using a mixture of 50:50 (*v/v*) ethanol–water [23,31,32,33].

The solubility of phenolic compounds in the solvent affects the extraction of these phytochemicals from plant matrices, with the solvent’s polarity being crucial for raising phenolics solubility [23]. Binary solvent systems are more effective for extracting phenolic compounds from plant materials compared to water or pure ethanol [30]. The intermediate polarity of these hydroalcoholic mixtures, similar to that of phenolic compounds, increases solubility, and consequently, the extraction yields of these compounds [33,34].

Moreover, water causes the plant material to swell, enhancing the contact surface area between the solid material and the solvent, whereas ethanol breaks the bonds between phenolic compounds and the plant matrix [35]. Ethanol is important in breaking hydrogen bonds and hydrophobic interactions between phenolics–proteins and phenolics–cellulose in the water–ethanol system. Increases in ethanol concentration can accelerate damage to the cell membranes of the plant matrix. Conversely, after a certain ethanol concentration and the consequent change in solvent polarity, the denaturation of proteins may occur. This promotes a faster extraction of impurities and hinders the diffusion of phenolic compounds [32,36].

**Figure 2:**
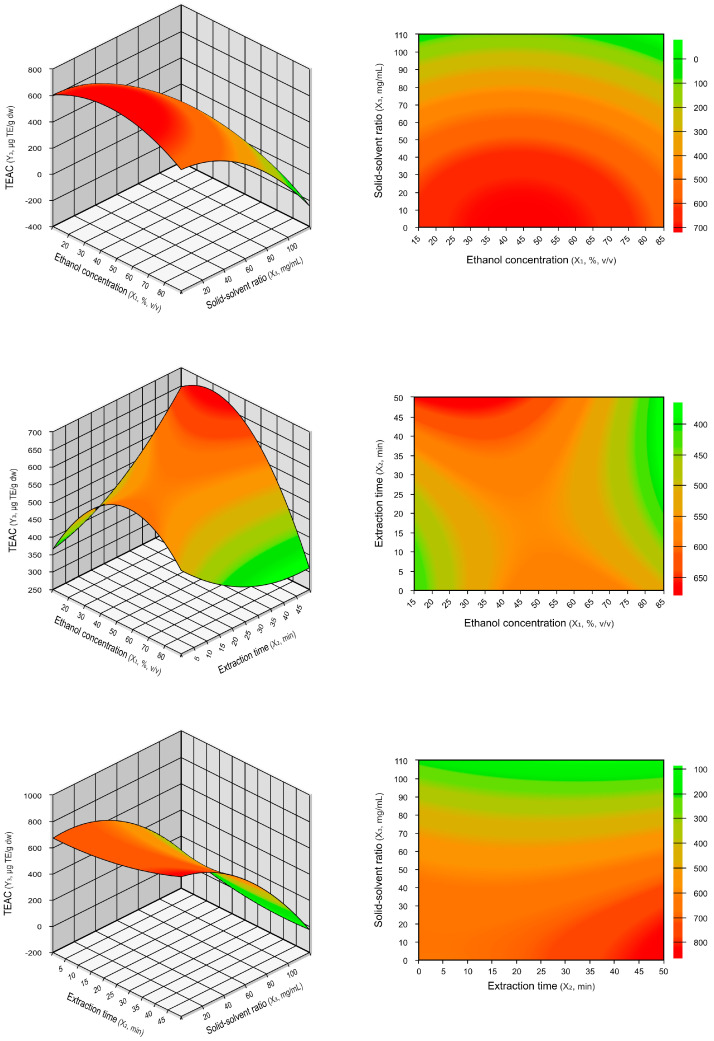
Three−dimensional surface and contour plots illustrating the interaction effects on TEAC based on ethanol concentration (%, *v/v*), extraction time (min), and solid–solvent ratio (mg/mL).

Although ethanol concentration was not statistically significant for TEAC (Table 2), the three-dimensional surface and contour plots of araticum peel extract for TEAC (Figure 2) indicate that the optimal values are close to 50% ethanol concentration, similar to what was previously reported for TPCs. Additionally, Figure 2 shows that TEAC significantly increased with a reduction in the solid–solvent ratio. This was due to the negative linear effect observed for the solid–solvent ratio, as the results in Table 2 corroborated.

The solid–solvent ratio is important because the diffusion mechanism depends on the difference in concentrations between the matrix and the solution [37]. Haas et al. [38] report that a larger solvent volume can extract more phenolic compounds due to the principles of mass transfer. In this context, the diffusion rate is directly proportional to the concentration gradient, which increases with a lower solid–solvent ratio [39]. Therefore, the lowest solid–solvent ratio investigated (10 mg/mL) for araticum peel allowed the extraction of more antioxidant compounds with maximum activity by the TEAC method. This analysis measures the ability of antioxidants in the sample to reduce the ABTS•^+^ radical cation by transferring electrons or hydrogen atoms [40]. It is suitable for lipophilic and hydrophilic antioxidants and presents a strong correlation with the biological activity of antioxidants, so it is widely used to determine the antioxidant capacity of phenolic compounds [41].

The type of solvent, extraction time, and solid–solvent ratio have a significant impact on the extraction process and efficiency [24]. Thus, optimizing extraction parameters is important for reducing extraction time, organic solvent consumption, energy expenditure, and costs. It is essential to identify the shortest possible extraction time without compromising the maximum recovery of phenolic compounds. Notwithstanding, the extraction time must be enough for the solvent to penetrate the plant tissue, dissolving the target compounds and diffusing them into the extraction medium [42].

A longer contact time between the solvent and the plant matrix can progressively increase the release of solutes from the matrix to the solvent [23]. However, according to Fick’s second law, the solute extraction rate increases until the equilibrium state is reached between the concentration in the plant matrix and the surrounding solvent, after which the compound extraction becomes constant or decreases because of degradation [23,43]. In this study, there was no significant difference (*p* > 0.05) in the effect of time (Table 2) for both response variables (TPCs and TEAC), suggesting that the antioxidant phenolic compounds present in araticum peel diffuse rapidly into the extracting solvent. For this reason, the shortest time (5 min) was considered the optimal condition for extraction.

#### 2.1.2. Optimization and Validation of Extraction Parameters

The optimal extraction conditions for TPCs and TEAC were determined by analyzing the generated mathematical models (Equations (1) and (2)) and the response surface plots (Figure 1 and Figure 2). A validation test was performed to verify the models’ reliability, and the results are presented in Table 4. The optimal extraction values predicted by the regression models were 68.47 mg GAE/g dw for TPCs and 677.04 µmol TE/g dw for TEAC. These values were significantly equal (*p* ≤ 0.05) to the experimental values obtained under the actual operating conditions of 50% ethanol concentration, 5 min extraction time, and 10 mg/mL solid–solvent ratio. The relative standard errors (RSEs) observed between the experimental and predicted values for TPCs and TEAC were low, at 2.41% and −1.47%, respectively. The relative standard errors measure the percentage deviation between the experimental values and the values predicted by the model [23]. These results indicate the accuracy of the obtained polynomial regression models and the precision of the optimal extraction conditions.

### 2.2. Chemical Characterization of Araticum Peel Extract Obtained under Optimized Conditions

#### 2.2.1. Determination of TPCs, CTs, and Antioxidant Activity

The results for the total phenolic compounds (TPC), condensed tannins (CTs), and antioxidant activity of the araticum peel in the optimized extraction conditions are shown in Table 5. TPCs results were obtained using the Folin–Ciocalteu assay, a reference method to determine and quantify total phenolic compounds in a variety of foods due to its simplicity and reproducibility [44]. The TPCs extracted from araticum peel was 70.16 mg GAE/g dw (43.52 mg GAE/g fresh weight (fw)). Fruits can be classified into three different categories according to TPCs: low (<1 mg GAE/g fw), intermediate (1–5 mg GAE/g fw), and high (>5 mg GAE/g fw) [3]. Following this classification, araticum peel has a high phenolic compound content and can be considered a source of these phytochemicals.

A similar result was observed by Arruda et al. [10] who obtained a maximum TPCs value of 70.68 mg GAE/g dw in araticum peel using a hydroethanolic solution (50% ethanol, *v/v*) and ultrasound-assisted extraction. Lesser TPCs results have also been reported. For example, another study by Arruda et al. [5], extracting free, esterified, glycosylated, and insoluble-bound phenolic fractions of araticum peel, obtained a total value maximum of 31.65 mg GAE/g dw, while Ramos et al. [9] found TPCs values varying from 8.34 to 19.27 mg GAE/g fw in araticum peel from various cities in the state of Minas Gerais.

Condensed tannins or proanthocyanidins are a class of phenolics with high antioxidant activity, formed by the condensation flavan-3-ol structural units [41]. The tannins participate in the defense of plants against UV radiation and attacks by herbivores, fungi, and viruses, thus tending to concentrate in the outermost layer of fruit. For this reason, fruit peels tend to have a high amount of condensed tannins [5]. This study demonstrated that araticum peel had an elevated content of condensed tannins, with 76.49 mg CE/g dw. In comparison, the study by Arruda et al. [5] showed a considerably lower content, of only 17.96 mg CE/g dw, considering the total of the different fractions of araticum peel extract evaluated.

Phenolic compounds are important for human health due to their antioxidant capacity as they can react with reactive species through three mechanisms: (1) hydrogen atom transfer or (2) electron donation inactivating free radicals, and (3) metal ion chelation forming stable complexes. These abilities of phenolic compounds can prevent or mitigate oxidative stress-related diseases, such as inflammation, cancer, diabetes, and cardiovascular diseases [41,45].

The antioxidant capacity of plant extracts, measured by various methods, is associated with the concentration of phenolic compounds using the Folin–Ciocalteu method [44]. In this study, the antioxidant activity of araticum peel was determined using synthetic free radical scavenging methods (TEAC and FRAP) and reactive oxygen species (ROS) scavenging methods (Table 5). Comparing the values obtained by different assays, araticum peel showed a greater ability to scavenge peroxyl radicals (ROO•) (1596.05 µmol TE/g dw or 990.19 µmol TE/g fw), followed by FRAP (730.57 µmol TE/g dw or 453.25 µmol TE/g fw) and TEAC (667.22 µmol TE/g dw or 413.94 µmol TE/g fw), which had similar values. This indicates that the bioactive compounds in the araticum peel, particularly phenolic compounds, exercise antioxidant activity more efficiently by the mechanism of transfer of hydrogen atoms because FRAP and TEAC methods are based on electron transfer, specifically for the cationic ferric ion and ABTS•^+^ radical cation [46].

The TEAC result was similar to that reported in the study of Arruda et al. [10] who obtained a maximum value of 613.76 μmol TE/g dw. However, our results were superior to those reported in other studies for araticum peel: 292.28 μmol TE/g dw [5] and 95.52–367.63 μM TE/g fw [9] for TEAC; 7.18–273.63 μM TE/g fw for FRAP [9]; and 448.29 µmol TE/g dw [5] and 315.89–525.41 μmol TE/g dw [10] for peroxyl radical scavenging activity. The higher values of antioxidant activity observed in this study, compared to those reported in the literature, can be attributed to the optimized extraction conditions. On account of this, it is essential to optimize the extraction parameters to avoid underestimating results and to ensure a more accurate characterization of the extract. Moreover, the high antioxidant activity observed by the different methods evaluated may be related to the high phenolic compound content found in araticum peel as observed in the Folin–Ciocalteu method.

Differences in the TPCs, condensed tannins, and antioxidant activity reported in different studies for the same plant material are influenced not only by extraction factors (solid–solvent ratio, extraction time and temperature, solvent composition, particle size, solvent polarity, and pH) [45] but also by geographical and environmental conditions, such as temperature, soil, sunlight exposure, harvest season, as well as physiological and genetic factors of the plant [3].

In addition to ROO•, hydroxyl radical (•OH), hypochlorous acid (HOCl), and superoxide radical (•O_2_^−^) were also investigated in this study (Table 5). ROS are reactive molecules produced in biological systems from oxygen metabolism, responsible for important biological processes, including cellular redox homeostasis, signal transduction, and defense against pathogens. Nonetheless, in high levels and uncontrolled, they lead to oxidative stress and damage to essential biomolecules such as DNA, RNA, proteins, and membranes, which can result in cardiovascular, kidney, inflammatory, and neurodegenerative diseases, arthritis, and cancer [47,48,49].

In general, araticum peel was able to eliminate all ROS studied. Considering that IC_50_ (inhibitory concentration) represents the extract concentration required to reduce the oxidative effect of reactive species by 50%, the results in Table 5 show a greater scavenging activity for •OH, followed by HOCl and •O_2_^−^ (0.26, 40.40, and 89.23 µg/mL dw, respectively). This result is relevant because the hydroxyl radical (•OH) is one of the most reactive ROS. The •OH has an unpaired pair of electrons, which can react with oxygen in its ground triple state. Furthermore, this species can interact with all biological molecules, causing cellular damage to lipids, proteins, and membranes [50].

HOCl is also a highly oxidative species produced by H_2_O_2_-mediated chloride oxidation catalyzed by myeloperoxidase (MPO) [47]. HOCl acts as a defense mechanism against microorganisms phagocytosed by neutrophils, but in excess, it can cause serious tissue damage [51]. Although •O_2_^−^ is not a potent pro-oxidant reactive species, it is an important precursor for the formation of other reactive species, for example, hydroxyl radical (•OH), singlet oxygen (^1^O_2_), and hydrogen peroxide (H_2_O_2_) [50,52].

To the best of our knowledge, this is the first study reporting the capacity of the hydroethanolic extract (ethanol 50%, *v/v*) from araticum peel to scavenge ROS. The araticum peel proved capable of scavenging all ROS tested in this study, especially acting as a strong scavenger of •OH. This action is important to impede the excessive formation of ROS and damage to important biomolecules, preventing cell death and, consequently, the early aging of the organism and the development of chronic diseases. Thus, exploring araticum peel extract in developing healthy products represents a promising strategy to offer significant health benefits and assist in preventing diseases associated with oxidative stress.

#### 2.2.2. Identification and Quantification of Phenolic Compounds by HPLC-DAD

The results of the HPLC-DAD analysis, performed to characterize the phytochemical profile of the optimized extract from araticum peel, are presented in Table 6. A total of 33 phenolic compounds were investigated, out of which 12 were identified and quantified. The main class of phenolic compounds in araticum peel was flavonoids, with a concentration of 6043.01 µg/g dw, representing 97.77% of the total identified compounds.

According to Jucá et al. [53], flavonoids can inhibit several ROS, such as superoxide and hydroxyl radicals. Phenolic acids possess hydroxyl and methoxyl groups on the aromatic ring, which enable effective scavenging of free radicals [41]. The antioxidant activity of an extract depends not only on the phenolic content but also on its chemical characteristics (number of aromatic and hydroxyl groups, substituent groups, specific positioning of these groups, and glycosylation degree) [3]. Additionally, flavonoids can interact synergistically with phenolic acids, enhancing antioxidant activity [10]. This fact may explain the high antioxidant capacity of araticum peel, mainly in the inhibition of ROS observed in this study.

Within the class of flavonoids, the major compounds found, in descending order, were as follows: procyanidin B2, epicatechin, catechin, and rutin (3248.77, 2526.12, 100.96, and 79.10 µg/g dw, respectively). For phenolic acids, the main compounds were vanillic acid, chlorogenic acid, and α-resorcylic acid (61.15, 29.04, and 28.16 µg/g dw, respectively). Other studies have also identified a similar phytochemical profile, with flavonoids being the predominant phenolics in araticum peel. The main flavonoids found by Justino et al. [8] were procyanidins B2 and C1, epicatechin, catechin, and quercetin. Ramos et al. [9] identified similar compounds, such as procyanidins A2 and B3, epicatechin, and quercetin, while the study by Arruda et al. [5] evidenced catechin, epicatechin, and quercetin (3526.78, 1632.90, and 21.83 µg/g dw, respectively) as the major flavonoids; while protocatechuic acid and caffeic acid (317.90 µg/g dw and 93.45 µg/g dw, respectively) were the main phenolic acids for araticum peel.

Procyanidin B2 was the most abundant phenolic compound in the araticum peel followed by epicatechin, with concentrations of 3248.77 and 2526.12 µg/g dw, respectively. Procyanidin B2 and epicatechin represent 93.43% of the total phenolic compounds identified in araticum peel. The high concentration of these compounds may explain the high condensed tannins value presented in Table 5.

Procyanidins are oligomers or polymers of flavan-3-ols, also known as catechin monomers [41]. Procyanidin B2, epicatechin, and catechin were the main flavan-3-ols found in the araticum peel, comprising 95.06% of the total phenolic compounds identified. Procyanidins can activate the Nrf2 pathway or inhibit the MAPK/NF-κB pathway, scavenge hydroxyl and superoxide radicals, and upregulate endogenous antioxidant enzymes [14,54]. Besides, they exhibit diverse biological activities, including anti-inflammatory, antidiabetic, anticancer effects, and cardiovascular and neural protective properties [55,56].

The analysis of araticum peel extract highlights its rich flavonoid content, particularly procyanidin B2, epicatechin, and catechin, which comprised most of its phenolic content. These compounds are likely responsible for the potent antioxidant properties of araticum peel, effectively neutralizing reactive oxygen species. These findings underscore the potential of araticum peel as an exceptional source of phenolics for food, cosmetic, and nutraceutical applications and contribute to the development of future research to elucidate potential health benefits.

## 3. Materials and Methods

### 3.1. Chemicals and Reagents

Folin–Ciocalteu reagent, fluorescein, Trolox (6-hydroxy-2,5,7,8-tetramethylchroman-2-carboxylic acid), AAPH [2,2′-azobis(2-methylamidinopropane)-dihydrochloride], ABTS [2,2′-azinobis-(3-ethylbenzothiazoline-6-sulfonic acid)-diammonium salt], TPTZ [2,4,6-Tris(2-pyridyl)-s-triazine], acetonitrile and formic acid grade HPLC, the standards with a purity of ≥96% (apigenin, apigetrin, astragalin, gallic acid, procyanidins (A2, B1, and B2), protocatechuic acid, catechin, chlorogenic acid, 4-hydroxybenzoic acid, benzoic acid, epicatechin, caffeic acid, gentisic acid, vanillic acid, trans-cinnamic acid, p-coumaric acid, vitexin, sinapic acid, syringic acid, ferulic acid, α-resorcylic acid, naringenin, rutin, myricetin, quercetin, quercetrin, luteolin, hyperoside, hesperetin, and kaempferol) were purchased from Sigma-Aldrich Chemical Co.^®^ (St. Louis, MO, USA).

### 3.2. Plant Material

Araticum fruits (*Annona crassiflora* Mart.) with full maturity were collected in Carmo do Paranaíba, Minas Gerais, Brazil (19°00′03″ south latitude, 46°18′58″ west longitude, and 1061 m altitude). A voucher specimen (UEC 197249) was deposited in the Herbarium of the Institute of Biology at the University of Campinas, Brazil (Herbarium UEC). Access to genetic heritage components (registration number A437549) was authorized by the Genetic Heritage Management Board (CGen) under Law n° 13.123/2015 and its related regulations. The fruits were washed and manually pulped. The obtained peel was freeze-dried (LIOTOP, model L101, São Carlos, SP, Brazil), ground using a knife grinder (Marconi, model MA340, Piracicaba, SP, Brazil), and stored at −20 °C until further analysis.

### 3.3. Ultrasound-Assisted Extraction

Freeze-dried araticum peel (from 50–500 mg depending on the solid–solvent ratio used in each experimental run, as can be seen in Table 1) was placed into a centrifuge tube and 5 mL of the hydroethanolic solution was added. These mixtures were homogenized for one minute and subjected to ultrasound-assisted extraction in an ultrasonic bath (UNIQUE, model UCS-2850A, 25 kHz, 120 W, São Paulo, SP, Brazil). The ethanol concentration (20–80% *v/v*), extraction time (5–45 min), and solid–solvent ratio (10–100 mg/mL) were predetermined according to the experimental design shown in Table 1. After extraction, the extracts were centrifuged at 4000 ×*g* for 25 min at 5 °C, and the upper layers were collected and stored at −20 °C until analysis.

### 3.4. Experimental Design

Effects of the independent variables in the extraction process using an ultrasound bath included ethanol concentration (20–80%, *v/v*), extraction time (5–45 min), and solid–solvent ratio (10–100 mg/mL) on the TPCs and antioxidant activity (TEAC) from araticum peel were investigated using a central composite rotatable design (CCRD). The experimental design comprised three central points, six axial points, and eight factorial points, totaling 17 experiments, generated using the software Protimiza Experimental Design^®^, online version (https://experimental-design.protimiza.com.br/, accessed on 8 September 2024, Protimiza, Campinas, SP, Brazil). The TEAC assay evaluates the capacity of antioxidants in the samples to reduce the ABTS•^+^ radical cation by an electron or hydrogen atom transfer. This method was chosen because it is simple, reproducible, and allows the quantification of hydrophilic and lipophilic antioxidants [57,58]. The independent variables and their ranges were determined according to previous studies [23,59,60]. The range and levels of the independent variables used in the coded and actual value form are given in Table 1. The experimental data were fitted to the second-order polynomial model as shown in Equation (3):

R = β_0_ + β_1_x_1_ + β_2_x_2_ + β_3_x_3_ + β_11_x_1_^2^ + β_22_x_2_^2^ + β_33_x_3_^2^ + β_12_x_1_x_2_ + β_13_x_1_x_3_ + β_23_x_2_x_3_ (3)

where R is the measured predicted response; β_0_ is the intercept; β_1_, β_2_, and β_3_ represent the linear coefficients; β_11_, β_22_, and β_33_ signify the quadratic coefficients; β_12_, β_13_, and β_23_ imply the interaction coefficients; and x_1_, x_2_ and x_3_ are the coded values of the independent variables.

The three-dimensional response surface plot and contour plot were based on the model regression coefficients. These plots illustrated the relationship between the response and the levels of each independent variable, aiding in determining optimal extraction conditions. The statistical significance of the regression coefficients was determined using Student’s *t*-test, and the polynomial regression model was evaluated using ANOVA, both in the Protimiza Experimental Design^®^, online version (Protimiza, Campinas, SP, Brazil).

The Student’s *t*-test was also used to analyze the difference between experimental and predicted values using Minitab software version 18.1 (Minitab Inc., State College, PA, USA). All statistical analyses were conducted at a 5% significance level (*p* ≤ 0.05).

### 3.5. Determination of Total Phenolic Compounds (TPCs)

Total phenolic compounds were determined using the method described by Roesler et al. [61] with modifications. Briefly, 30 μL of diluted extract was mixed with 150 μL of 10% (*v/v*) Folin–Ciocalteu reagent and 120 μL of 7.5% (*w/v*) sodium carbonate solution and then incubated for 6 min at 45 °C. The absorbance was measured at 760 nm against a blank on a microplate reader (SPECTROstar Nano, BMG Labtech, Ortenberg, Germany). Gallic acid was used as a standard and the results were expressed as milligrams of gallic acid equivalents per gram of dried peel (mg GAE/g dw). This analysis was independently performed three times and in three replicates. Data are presented as the mean ± standard deviation.

### 3.6. Determination of Condensed Tannins (CTs) Content

Condensed tannins content was determined according to Arruda et al. [5] with alterations. The diluted extract (20 μL) was mixed with 4% (*w/v*) vanillin in methanol (180 μL) and concentrated HCl (90 μL). Then, the reaction mixture was incubated for 20 min at room temperature and the absorbance was recorded at 500 nm using a microplate reader (SPECTROstar Nano, BMG Labtech, Ortenberg, Germany). Catechin was used for the standard curve and the results were expressed as milligrams of catechin equivalents per gram of dried peel (mg CE/g dw). This analysis was independently performed three times and in three replicates. Data are presented as the mean ± standard deviation.

### 3.7. Antioxidant Activity

#### 3.7.1. Ferric Reducing Antioxidant Power (FRAP) Assay

The FRAP assay was conducted according to Benzie and Strain et al. [62] with modifications. FRAP solution was produced by mixing 0.3 mol/L acetate buffer at pH 3.6, 10 mmol/L TPTZ, and 20 mmol/L ferric chloride in a ratio of 10:1:1 (*v/v/v*). The diluted extract (20 µL), FRAP solution (180 µL), and deionized water (60 µL) were combined and incubated at 37 °C for 30 min. Then, the absorbance was read at 595 nm using a microplate reader (SPECTROstar Nano, BMG Labtech, Ortenberg, Germany). The results were expressed as micromoles of Trolox equivalents per gram of dried peel (μmol TE/g dw). This assay was independently performed three times and in three replicates. Data are presented as the mean ± standard deviation.

#### 3.7.2. Trolox Equivalent Antioxidant Capacity (TEAC) Assay

The TEAC was determined based on the method described by Re et al. [40] with modifications. At first, the ABTS•^+^ radical cation was generated by mixing 5 mL of 7 mmol/L ABTS and 88 μL of 140 mmol/L potassium persulfate and allowed to stand at room temperature overnight. Subsequently, the ABTS•^+^ working solution was diluted with ultrapure water until reaching the absorbance of 0.70 ± 0.02 at 734 nm. Extract aliquots (50 μL) and ABTS•^+^ work solution (250 μL) were mixed and incubated for 6 min at room temperature before measuring the absorbance at 734 nm using a microplate reader (SPECTROstar Nano, BMG Labtech, Ortenberg, Germany). The results were expressed as micromoles of Trolox equivalents per gram of dried peel (μmol TE/g dw). This assay was independently performed three times and in three replicates. Data are presented as the mean ± standard deviation.

#### 3.7.3. Peroxyl Radical (ROO•) Scavenging Activity

The peroxyl radical scavenging activity was assessed by observing the effect of the extract on the delay of fluorescence decay peroxyl radical-induced fluorescein oxidation following the method described by Saliba et al. [63]. The fluorescein (508.25 nmol/L) and AAPH (76 mmol/L) solutions were prepared in 75 mmol/L potassium phosphate buffer at pH 7.4. Aliquots of different concentrations of the extract (20 µL) were mixed with fluorescein (60 µL) and AAPH (110 µL) solutions. The fluorescence was read in a microplate reader (Molecular Devices, LLC, Sunnyvale, CA, USA) at 37 °C at the kinetic mode (readings every minute for 120 min) with wavelengths of emission and excitation of 528 and 485 nm, respectively. The results were expressed as micromoles of Trolox equivalents per gram of dried peel (μmol TE/g dw). This assay was independently performed three times and in three replicates. Data are presented as the mean ± standard deviation.

#### 3.7.4. Hydroxyl Radical (•OH) Scavenging Activity

The hydroxyl radical scavenging activity was monitored by the increase in luminescence resulting from luminol oxidation according to Mariutti et al. [64] with modifications. The reaction system was formed by the addition of 50 µL of carbonate buffer (0.5 mol/L, pH 10), 50 µL of extract (different concentrations), 50 µL of 100 µmol/L luminol solution prepared in the carbonate buffer (0.5 mol/L, pH 10), 50 µL of FeCl_2_-EDTA solution (125 e 500 µmol/L), and 50 µL of H_2_O_2_ solution (17.5 mmol/L). Luminescence was read at 37 °C using a microplate reader (Molecular Devices, LLC, Sunnyvale, CA, USA) after 5 min of incubation. The results were expressed as IC_50_ (μg/mL dw). This assay was independently performed three times and in three replicates. Data are presented as the mean ± standard deviation.

#### 3.7.5. Hypochlorous Acid (HOCl) Scavenging Activity

The hypochlorous acid scavenging activity was carried out by monitoring the effects of the extract on the reduction of HOCl-induced dihydrorhodamine 123 (DHR) oxidation following the method described by Saliba et al. [63]. HOCl was generated after adjusting the pH of a 1% NaOCl solution to 6.2 with 10% H_2_SO_4_ solution. The concentration of HOCl solution was corrected by diluting in phosphate buffer (100 mmol/L, pH 7.4) and was measured at 235 nm, using the molar absorption coefficient 100 M^−1^cm^−1^. The DHR was diluted at a concentration of 1.25 µmol/L in the phosphate buffer (100 mmol/L, pH 7.4), immediately before the analysis. Different extract concentrations (100 μL), phosphate buffer (100 μL, pH 7.4), DHR (50 μL), and 5 μmol/L HOCl (50 μL) were mixed, and the fluorescence was read at 37 °C in a microplate reader (Molecular Devices, LLC, Sunnyvale, CA, USA) using wavelengths of emission of 528 nm and excitation of 485 nm. The results were expressed as IC_50_ (μg/mL dw). This assay was independently performed three times and in three replicates. Data are presented as the mean ± standard deviation.

#### 3.7.6. Superoxide Radical (•O_2_^−^) Scavenging Activity

Superoxide radical scavenging activity was assessed by monitoring the effect of the extract on the superoxide radical-induced nitroblue tetrazolium (NBT) reduction. The •O_2_^−^ was produced by the non-enzymatic nicotinamide adenine dinucleotide hydrate (NADH)/phenazine methosulfate (PMS) system [51]. Briefly, 166 µmol/L NADH (100 μL), 107.5 µmol/L NBT (50 μL), 2.7 µmol/L PMS (50 μL), and different extract concentrations (100 μL) were dissolved in potassium phosphate buffer solution (19 mmol/L, pH 7.4) and incubated in the dark for 5 min before reading the absorbance at 560 nm in the microplate reader (Molecular Devices, LLC, Sunnyvale, CA, USA). The results were expressed as IC_50_ (μg/mL dw). This assay was independently performed three times and in three replicates. Data are presented as the mean ± standard deviation.

### 3.8. HPLC-DAD Analysis of Phenolic Compounds

The phenolic compounds determination in araticum peel extract was performed by using a Dionex UltiMate 3000 system coupled to a diode array detector (Thermo Fisher Scientific, Waltham, MA, USA), according to Silva et al. [58]. Chromatographic separation was conducted on a reverse-phase AcclaimTM 120 A C18 column (250 × 4.6 mm i.d., 5 μm particle size, Thermo Fisher Scientific, Waltham, MA, USA) using a gradient elution. The column oven, injection volume, and flow rate were set at 32 °C, 20 μL, and 0.5 mL/min, respectively. The elution solvents were 0.1% formic acid in deionized water (Eluent A) and HPLC-grade acetonitrile (Eluent B). The phenolic compounds were eluted according to the following gradient (only B % values are presented, and the rest of the mobile phase composition consisted of eluent A): 0–5 min, 5% B; 5–27 min, 5–29% B; 27–33 min, 35% B; 33–45 min, 35–50% B; 45–50 min, 95% B; and 50–60 min, 5% B. The UV-Vis absorption spectra of the standards and samples were measured between 190 and 800 nm. Phenolic compounds were identified by comparing their retention times and UV-Vis absorbance spectra with those of authentic standards and quantified at different wavelengths depending on the compound (260, 280, 320, or 360 nm). The content of phenolic compounds was expressed as micrograms per gram of dried peel (µg/g dw). This analysis was independently performed in three replicates. Data are presented as the mean ± standard deviation.

## 4. Conclusions

Hydroethanolic solutions coupled with ultrasound-assisted extraction were used as a green, simple, fast, and effective method for extracting phenolic compounds and antioxidants from araticum peel. This study effectively applied response surface methodology to optimize the ultrasound-assisted extraction conditions to maximize the TPCs and TEAC. The optimal extraction conditions were 50% (*v/v*) of ethanol concentration, 5 min of extraction time, and 10 mg/mL solid–solvent ratio. Under these extraction conditions, the experimental values for the TPCs and TEAC were 70.16 mg GAE/g dw and 667.22 µmol TE/g dw, respectively, which were significantly equivalent to the predicted values by the mathematical model (68.47 mg GAE/g dw and 677.04 µmol TE/g dw, respectively). In addition to the significant amount of TPCs, the araticum peel showed a high condensed tannins content. The phytochemical profile by HPLC-DAD revealed the presence of 12 phenolic compounds, with flavonoids being the main class identified (97.77%), highlighting procyanidin B2 (3248.77 μg/g dw), epicatechin (2526.12 μg/g dw), and catechin (100.96 μg/g dw) as the major compounds. The presence of these phenolics may probably be the reason for the high antioxidant activity observed against synthetic free radicals and ROS, especially in scavenging peroxyl and hydroxyl radicals, which could prevent or alleviate diseases related to oxidative damage. Therefore, this experimental study demonstrates that it was possible to recover significant amounts of phenolic compounds with antioxidant activity from araticum peel, offering a promising opportunity for managing plant matrix byproducts using greener technology. This extract and its phenolic compounds can be explored in future research for applications in the food, cosmetic, and pharmaceutical industries, aimed at developing functional foods, active food packaging, edible films, nutraceuticals, cosmetics, personal hygiene products, and medications to prevent aging, cancer, and inflammation, among and other oxidative stress-related diseases.

## Figures and Tables

**Table 1:** Experimental design and response variables for TPCs and TEAC in araticum peel.

Run	Coded Values			Actual Values			Response Variables	
	EC	ET	SSR	EC (%, *v/v*)	ET (min)	SSR (mg/mL)	TPCs (mg GAE/g dw)	TEAC (µmol TE/g dw)
	x_1_	x_2_	x_3_	X_1_	X_2_	X_3_	R_1_	R_2_
1	−1.00	−1.00	−1.00	32.14	13.10	28.21	62.71 ± 0.88	654.56 ± 21.37
2	1.00	−1.00	−1.00	67.86	13.10	28.21	69.91 ± 0.99	724.31 ± 21.06
3	−1.00	1.00	−1.00	32.14	36.90	28.21	74.48 ± 3.25	832.43 ± 6.04
4	1.00	1.00	−1.00	67.86	36.90	28.21	59.66 ± 2.73	661.53 ± 18.04
5	−1.00	−1.00	1.00	32.14	13.10	81.79	56.61 ± 0.88	354.82 ± 12.36
6	1.00	−1.00	1.00	67.86	13.10	81.79	51.68 ± 1.65	270.04 ± 9.34
7	−1.00	1.00	1.00	32.14	36.90	81.79	62.66 ± 0.62	323.33 ± 14.31
8	1.00	1.00	1.00	67.86	36.90	81.79	69.21 ± 3.10	285.06 ± 5.10
9	−1.68	0.00	0.00	19.97	25.00	55.00	53.83 ± 0.47	486.02 ± 14.10
10	1.68	0.00	0.00	80.03	25.00	55.00	50.34 ± 1.82	426.59 ± 8.39
11	0.00	−1.68	0.00	50.00	4.98	55.00	62.04 ± 1.76	555.54 ± 7.19
12	0.00	1.68	0.00	50.00	45.02	55.00	65.92 ± 3.45	597.68 ± 11.35
13	0.00	0.00	−1.68	50.00	25.00	9.95	59.94 ± 2.24	570.53 ± 13.67
14	0.00	0.00	1.68	50.00	25.00	100.05	59.39 ± 1.82	316.99 ± 5.36
15	0.00	0.00	0.00	50.00	25.00	55.00	68.62 ± 0.76	520.96 ± 17.33
16	0.00	0.00	0.00	50.00	25.00	55.00	69.14 ± 1.53	583.27 ± 12.15
17	0.00	0.00	0.00	50.00	25.00	55.00	69.02 ± 0.75	576.79 ± 14.51

dw: dry weight; EC: ethanol concentration; ET: extraction time; GAE: gallic acid equivalents; SSR: solid–solvent ratio; TPCs: total phenolic compounds; TEAC: Trolox equivalent antioxidant capacity; TE: Trolox equivalents.

**Table 2:** Regression coefficients and analysis of the CCRD model for the TPCs and TEAC in araticum peel.

Model Components	TPCs				TEAC			
	RC	SE	t-Value	*p*-Value	RC	SE	t-Value	*p*-Value
Mean	68.58	3.65	18.81	<0.0001 *	558.72	57.89	9.65	<0.0001 *
x_1_	−0.87	1.71	−0.51	0.6274	−23.74	27.19	−0.87	0.4116
x_2_	2.32	1.71	1.35	0.2184	12.41	27.19	0.46	0.6619
x_3_	−2.02	1.71	−1.18	0.2777	−151.28	27.19	−5.56	0.0008 *
x_1_^2^	−4.75	1.88	−2.52	0.0397 *	−31.19	29.92	−1.04	0.3318
x_2_^2^	−0.55	1.88	−0.29	0.7803	11.34	29.92	0.38	0.7160
x_3_^2^	−2.07	1.88	−1.10	0.3080	−35.63	29.92	−1.19	0.2726
x_1_x_2_	−1.32	2.24	−0.59	0.5745	−24.27	35.52	−0.68	0.5165
x_1_x_3_	1.16	2.24	0.52	0.6216	−2.74	35.52	−0.08	0.9407
x_2_x_3_	2.76	2.24	1.23	0.2576	−16.45	35.52	−0.46	0.6574

RC: regression coefficient; SE: standard error; TPCs: total phenolic compounds; TEAC: Trolox equivalent antioxidant capacity. * Significant statistical differences (*p* ≤ 0.05).

**Table 3:** ANOVA of second-order polynomial models for optimization of TPCs and TEAC parameters from araticum peel.

Source	TPCs					TEAC				
	SS	DF	MS	F-Value	*p*-Value	SS	DF	MS	F-Value	*p*-Value
Model	497.79	9.00	55.31	1.38	0.3428	358,061.73	9.00	39,784.64	3.94	0.0421 *
Residual	280.37	7.00	40.05			70,660.66	7.00	10,094.38		
Lack of fit	280.22	5.00	56.04	756.00	0.0013 *	68,313.49	5.00	13,662.70	11.64	0.0810
Pure error	0.15	2.00	0.07			2347.17	2.00	1173.59		
Total	778.16	16.00				428,722.39	16.00			
R^2^	0.6397					0.8352				

DF: degree of freedom; MS: mean squares; SS: sum of squares; TPCs: total phenolic compounds; TEAC: Trolox equivalent antioxidant capacity. * Significant statistical differences (*p* ≤ 0.05).

**Table 4:** Predicted and experimental values of TPCs and TEAC parameters from araticum peel.

Parameters		TPCs (mg GAE/g dw)			TEAC (µmol TE/g dw)		
	Optimum Conditions	PV	EV	RSE (%)	PV	EV	RSE (%)
EC (%, *v/v*)	50	68.47 a	70.16 ± 2.34 a	2.41	677.04 a	667.22 ± 42.24 a	−1.47
ET (min)	5						
SSR (mg/mL)	10						

dw: dry weight; EC: ethanol concentration; ET: extraction time; EV: experimental value; GAE: gallic acid equivalents; PV: predicted value; RSE: relative standard error; SSR: solid–solvent ratio; TPCs: total phenolic compounds; TEAC: Trolox equivalent antioxidant capacity; TE: Trolox equivalents. The same characters depict the insignificant statistical difference between the experimental and predicted values by Student’s *t*-test at 95% confidence. RSE (%) = (Experimental Value − Predicted Value) × 100/Experimental Value.

**Table 5:** Total phenolic compounds, condensed tannins, and antioxidant activities in araticum peel obtained under optimal extraction conditions.

Analysis	Parameters	Araticum Peel
Phytochemicals	TPCs (mg GAE/g dw)	70.16 ± 2.34
	CTs (mg CE/g dw)	76.49 ± 1.61
Synthetic free radical	TEAC (µmol TE/g dw)	667.22 ± 42.24
	FRAP (µmol TE/g dw)	730.57 ± 26.82
ROS	ROO• (µmol TE/g dw)	1596.05 ± 40.43
	•OH (IC_50_ µg/mL dw)	0.26 ± 0.01
	HOCl (IC_50_ µg/mL dw)	40.40 ± 0.89
	•O_2_^−^ (IC_50_ µg/mL dw)	89.23 ± 9.84

CE: catechin equivalents; CTs: condensed tannins; dw: dry weight; FRAP: ferric reducing antioxidant power; GAE: gallic acid equivalents; HOCl: hypochlorous acid scavenging activity; IC_50_: extract concentration that resulted in a 50% reduction in radical concentration compared to the untreated control; •O_2_^−^: superoxide radical scavenging activity; •OH: hydroxyl radical scavenging activity; ROO•: peroxyl radical scavenging activity; ROS: reactive oxygen species; TPCs: total phenolic compounds; TEAC: Trolox equivalent antioxidant capacity; TE: Trolox equivalents.

**Table 6:** Phenolic compounds profile and content in araticum peel obtained under optimal extraction conditions.

Class	Compound	Content (µg/g dw)
Phenolic acids	4-Hydroxybenzoic acid	n.d.
	Benzoic acid	n.d.
	Caffeic acid	n.d.
	Chlorogenic acid (5-caffeoylquinic acid)	29.04 ± 0.30
	Ferulic acid	13.86 ± 0.37
	Gallic acid	n.d.
	Gentisic acid (2,5-dihydroxybenzoic acid)	n.d.
	p-Coumaric acid	5.52 ± 0.25
	Protocatechuic acid (3,4-dihydroxybenzoic acid)	n.d.
	Sinapic acid	n.d.
	Syringic acid	n.d.
	Trans-cinnamic acid	n.d.
	Vanillic acid	61.15 ± 1.13
	α-Resorcylic acid (3,5-dihydroxybenzoic acid)	28.16 ± 0.51
	**Total phenolic acids**	**137.73 ± 2.18**
Flavonoids	Apigenin	n.d.
	Apigetrin (apigenin-7-O-glucoside)	n.d.
	Astragalin (kaempferol-3-O-glucoside)	22.45 ± 0.48
	Catechin	100.96 ± 1.46
	Epicatechin	2526.12 ± 23.22
	Hesperetin	n.d.
	Hyperoside (quercetin-3-O-galactoside)	45.67 ± 1.42
	Kaempferol	n.d.
	Luteolin	n.d.
	Myricetin	n.d.
	Naringenin	n.d.
	Procyanidin A2	n.d.
	Procyanidin B1	n.d.
	Procyanidin B2	3248.77 ± 33.52
	Quercetin	n.d.
	Quercetrin (quercetin-3-O-rhamnoside)	19.94 ± 1.35
	Rutin (quercetin-3-O-rutinoside)	79.10 ± 1.27
	Vitexin (apigenin-8-C-glucoside)	n.d.
	Vitexin-2″-O-rhamnoside	n.d.
	**Total flavonoids**	**6043.01 ± 52.30**
	**Total phenolic compounds**	**6180.74 ± 52.96**

dw: dry weight, n.d.: not detected.

## Data Availability

The authors confirm that the data supporting the findings of this study are available within the article.
